# Tubeless uniportal VATS in thoracic surgery – Indications, ERAS pathways, and outcomes: A review

**DOI:** 10.17305/bb.2026.13644

**Published:** 2026-01-20

**Authors:** Bo Zhang, Xian-hua Ye, De-shuang Xiao

**Affiliations:** 1 Department of Thoracic surgery, The First People’s Hospital of Wenling, Taizhou University Affiliated Wenling Hospital, School of Medicine, Taizhou University, Wenling, Zhejiang, China; 2 Department of Anesthesiology, The First People’s Hospital of Wenling, Taizhou University Affiliated Wenling Hospital, School of Medicine, Taizhou University, Wenling, Zhejiang, China; 3 Department of General Surgery, The First People’s Hospital of Wenling, Taizhou University Affiliated Wenling Hospital, School of Medicine, Taizhou University, Wenling, Zhejiang, China

**Keywords:** Tubeless thoracic surgery, uniportal video-assisted thoracoscopic surgery, minimally invasive thoracic surgery, non-intubated anesthesia, chest drain-free surgery, enhanced recovery after surgery, ERAS

## Abstract

Tubeless uniportal video-assisted thoracoscopic surgery (VATS) is an innovative approach characterized by the use of non-intubated (spontaneous-breathing) anesthesia, the omission of routine postoperative chest drainage, and single-port access. This technique has gained traction in recent years for a variety of thoracic procedures. While practices reported in the literature may differ, this review primarily examines the combined non-intubated and drainless approach. This narrative review provides a comprehensive overview and critical analysis of its current clinical applications, including sympathectomy, pulmonary wedge resection, spontaneous pneumothorax, thymectomy, and early-stage lung cancer. It also addresses essential aspects of perioperative management and procedural indications within enhanced recovery-oriented pathways. A systematic literature search of PubMed, Embase, and Web of Science was conducted to identify pertinent studies published between January 2010 and April 2025. Current clinical reports indicate potential benefits such as reduced postoperative pain, shorter hospital stays, and accelerated recovery. However, the existing evidence largely stems from small, observational studies with varied methodologies, necessitating cautious interpretation. The broader implementation of this technique in more complex procedures depends on the establishment of standardized clinical pathways, the refinement of multidisciplinary perioperative strategies, and validation through multicenter prospective studies. Tubeless uniportal VATS shows promise as a significant advancement in function-preserving and recovery-oriented thoracic surgery.

## Introduction

As a surgical specialty closely linked to respiratory and circulatory physiology, thoracic surgery has consistently pursued the dual goals of minimizing trauma and preserving function [1]. The evolution from traditional thoracotomy to multi-port thoracoscopy, and now to the increasingly adopted uniportal video-assisted thoracoscopic surgery (VATS), has established minimally invasive techniques as the prevailing trend in thoracic surgical practice [2]. Concurrently, the ongoing development of enhanced recovery after surgery (ERAS) has further advanced efforts to reduce surgical trauma and expedite postoperative rehabilitation. This has fostered increasing interest in techniques that not only minimize intraoperative injury but also optimize physiological preservation and functional outcomes [3].

In this context, tubeless thoracoscopic surgery has emerged as a novel approach aimed at further reducing perioperative invasiveness. In its most widely accepted form, tubeless uniportal VATS is characterized by a combination of non-intubated (spontaneous-breathing) anesthesia and the omission of routine postoperative chest drainage, performed through a single-port approach [4]. In the existing literature, practices may vary, including non-intubated or drainless variants; in this review, the term “tubeless uniportal VATS” primarily refers to the combined non-intubated and drainless approach, while related variants are discussed where relevant. Adjunctive measures, such as opioid-sparing analgesia and avoidance of postoperative analgesic pumps, are frequently incorporated as part of enhanced recovery pathways but are not considered mandatory definitional components. Built upon the foundation of uniportal VATS, this technique represents a further refinement in minimally invasive thoracic surgery [5]. Initial applications have demonstrated favorable outcomes in relatively low-risk procedures, such as thoracic sympathectomy, spontaneous pneumothorax, and pulmonary wedge resection. More recently, exploratory uses in more complex interventions—such as thymectomy and early-stage lung cancer resection—have also been reported [6, 7].

Despite its potential, current research on tubeless techniques predominantly consists of single-procedure reports or small-scale case studies. There is a lack of comprehensive narrative syntheses encompassing its broader application, including key factors such as procedural indications, patient selection criteria, intraoperative risk management, and long-term outcomes. This limited evidence base impedes its widespread implementation, particularly in oncologic or technically demanding surgeries.

Therefore, this narrative review aims to comprehensively summarize and critically discuss the clinical application of tubeless uniportal thoracoscopic surgery across a spectrum of thoracic procedures, encompassing both benign and malignant conditions. Particular emphasis is placed on its clinical feasibility, advantages, limitations, and future prospects. We also propose a framework for its structured adoption, aligned with minimally invasive and ERAS principles, to inform both clinical practice and future research.

## Literature search strategy

To inform this narrative review, a literature search was conducted using the PubMed, Embase, and Web of Science databases to identify relevant studies on tubeless uniportal VATS published between January 2010 and April 2025. The search strategy included combinations of keywords such as “tubeless VATS,” “non-intubated thoracic surgery,” “uniportal thoracoscopy,” and “enhanced recovery after surgery.” The search focused on English-language articles reporting clinical applications, perioperative techniques, or outcomes. Relevant publications were reviewed at both the title/abstract and full-text levels to identify studies pertinent to this review’s scope. Case reports involving fewer than three patients, animal studies, and conference abstracts without full articles were excluded.

For this review, studies were deemed eligible if they involved uniportal VATS and reported the use of non-intubated (spontaneous-breathing) anesthesia and/or the omission of routine postoperative chest drainage. Procedures fulfilling both non-intubated and drainless criteria were classified as “completely tubeless,” which constituted the primary focus of this review. Studies adopting only one of these elements were categorized as related variants and discussed where relevant. Adjunctive perioperative measures, including analgesic strategies, were not used as eligibility criteria.

## Basic concepts and development history of tubeless uniportal thoracoscopic technology

### Definition and core concept of tubeless technology

**Figure 1.**
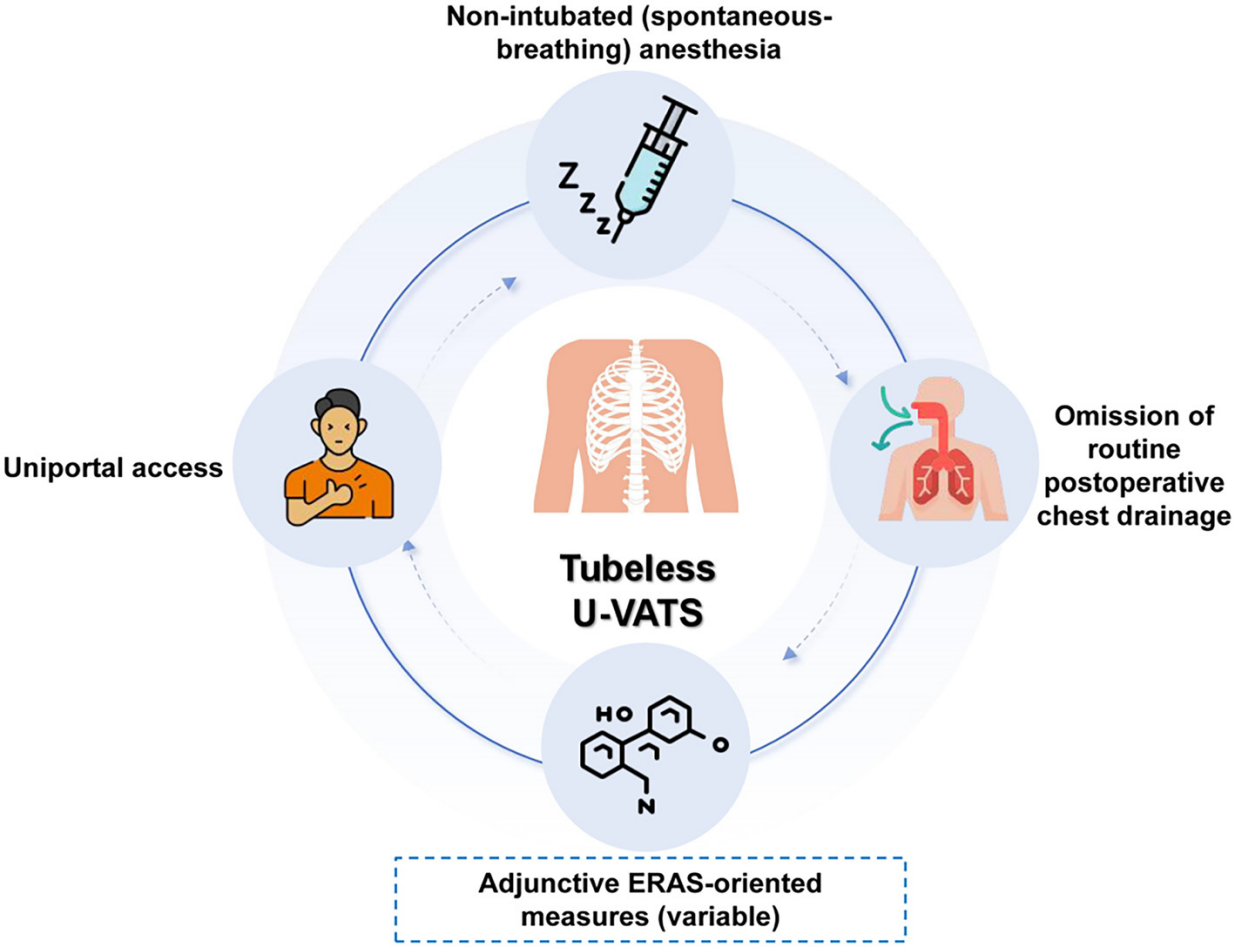
**Schematic overview of tubeless uniportal video-assisted thoracoscopic surgery (U-VATS) and adjunctive ERAS-oriented measures.** The tubeless U-VATS concept integrates uniportal access, non-intubated (spontaneous-breathing) anesthesia, and omission of routine postoperative chest drainage (core components). Adjunctive ERAS-oriented measures (variable; e.g., opioid-sparing analgesia and avoidance of postoperative analgesic pumps) may be added according to institutional protocols but are not mandatory definitional elements. Abbreviations: VATS: Video-assisted thoracoscopic surgery; U-VATS: Uniportal VATS; ERAS: Enhanced recovery after surgery.

Tubeless thoracoscopic surgery refers to a novel approach in thoracic surgery that aims to achieve a high level of minimal invasiveness by significantly reducing perioperative intervention [8]. In the context of this review, tubeless uniportal VATS is operationally defined by two mandatory core components: non-intubated (spontaneous-breathing) anesthesia and the omission of routine postoperative chest drainage, performed through a uniportal approach [9, 10). These two elements constitute the defining features used to classify and interpret the literature discussed in this review. Additional perioperative measures, such as opioid-sparing analgesia, avoidance of postoperative analgesic pumps, or other enhanced recovery–oriented strategies, are frequently adopted in clinical practice and are closely aligned with ERAS principles. However, these measures are not considered mandatory definitional components of tubeless uniportal VATS and were not used as inclusion criteria when summarizing published studies. An overview distinguishing core definitional components from adjunctive ERAS-related measures is illustrated in Figure 1. Beyond technical modifications, the tubeless concept reflects a broader shift in surgical philosophy toward functional preservation and enhanced recovery [11, 12]. Its conceptual foundation is closely linked to ERAS, which emphasizes minimizing surgical stress and preserving physiological function to facilitate early postoperative rehabilitation [13, 14].

The tubeless approach advocates for more than just smaller incisions—it promotes reduced physiological disruption and preservation of patient autonomy [15]. Compared with traditional intubation anesthesia, tubeless techniques preserve spontaneous respiration, decrease the risk of complications such as pharyngeal discomfort and bronchospasm, and potentially reduce alveolar damage and immune suppression associated with mechanical ventilation [16, 17]. Moreover, eliminating chest drainage tubes, together with ERAS-aligned analgesic strategies, may reduce postoperative pain, anxiety, and immobilization time, thereby contributing to a smoother and faster recovery process [18, 19]. In this context, tubeless surgery represents not only a technical innovation but also a patient-centered approach aimed at functional preservation and rapid rehabilitation.

### Evolution from traditional VATS to tubeless-uniportal approach

The development of tubeless technology is intricately linked to the ongoing evolution of thoracoscopic surgical techniques [20]. Initially, multiportal VATS required multiple incisions for instrument access, which represented a significant advancement in reducing surgical trauma. However, it was still associated with considerable postoperative pain and a heightened risk of intercostal nerve injury [21, 22]. The subsequent introduction of uniportal VATS allowed procedures to be performed through a single intercostal incision, enhancing visualization and instrument coordination. This approach has demonstrated clear benefits, including reduced pain, fewer complications, and improved patient satisfaction [23].

Despite the advancements of uniportal VATS, standard practices such as endotracheal intubation and routine postoperative chest drainage remain prevalent, limiting the potential for physiological preservation [24]. The tubeless uniportal approach was introduced to further minimize perioperative invasiveness by integrating non-intubated anesthesia and spontaneous ventilation with selective omission of postoperative chest drainage. This approach extends the minimally invasive advantages of uniportal VATS and has shown particular promise in low-complexity procedures such as pulmonary wedge resection and thoracic sympathectomy, where feasibility and safety have been demonstrated in early clinical experiences [25].

### Key enablers: progress in perioperative management

The successful implementation of tubeless uniportal thoracoscopic surgery relies on a comprehensive perioperative management strategy, encompassing anesthesia techniques, respiratory support, pain control, and postoperative care [7]. Non-intubated anesthesia is central to the tubeless concept. In most cases, total intravenous anesthesia (TIVA) combined with regional nerve blocks—such as thoracic paravertebral block (TPVB), intercostal nerve block (INB), or erector spinae plane block (ESPB)—is utilized to achieve adequate anesthesia and analgesia without requiring endotracheal intubation [26, 27]. Patients maintain spontaneous ventilation via high-flow nasal cannula (HFNC) or laryngeal mask airway, ensuring oxygenation while avoiding complications associated with positive pressure ventilation.

Effective perioperative analgesia is essential for supporting spontaneous breathing and early mobilization. In addition to regional nerve blocks, intraoperative infiltration with long-acting local anesthetics such as ropivacaine is commonly employed to reduce postoperative analgesic requirements [28]. Furthermore, the selective omission of chest drainage necessitates meticulous intraoperative hemostasis and careful assessment of air leakage. Surgeons must verify complete lung re-expansion and hemostatic stability before concluding the procedure [6]. Collectively, these strategies aim to maintain physiological stability and facilitate early recovery, thereby supporting the safe implementation of the “no intubation, no drain” paradigm within minimally invasive thoracic surgery.

## Current status of application of tubeless uniportal thoracoscopic surgery in different thoracic procedures

**Figure 2.**
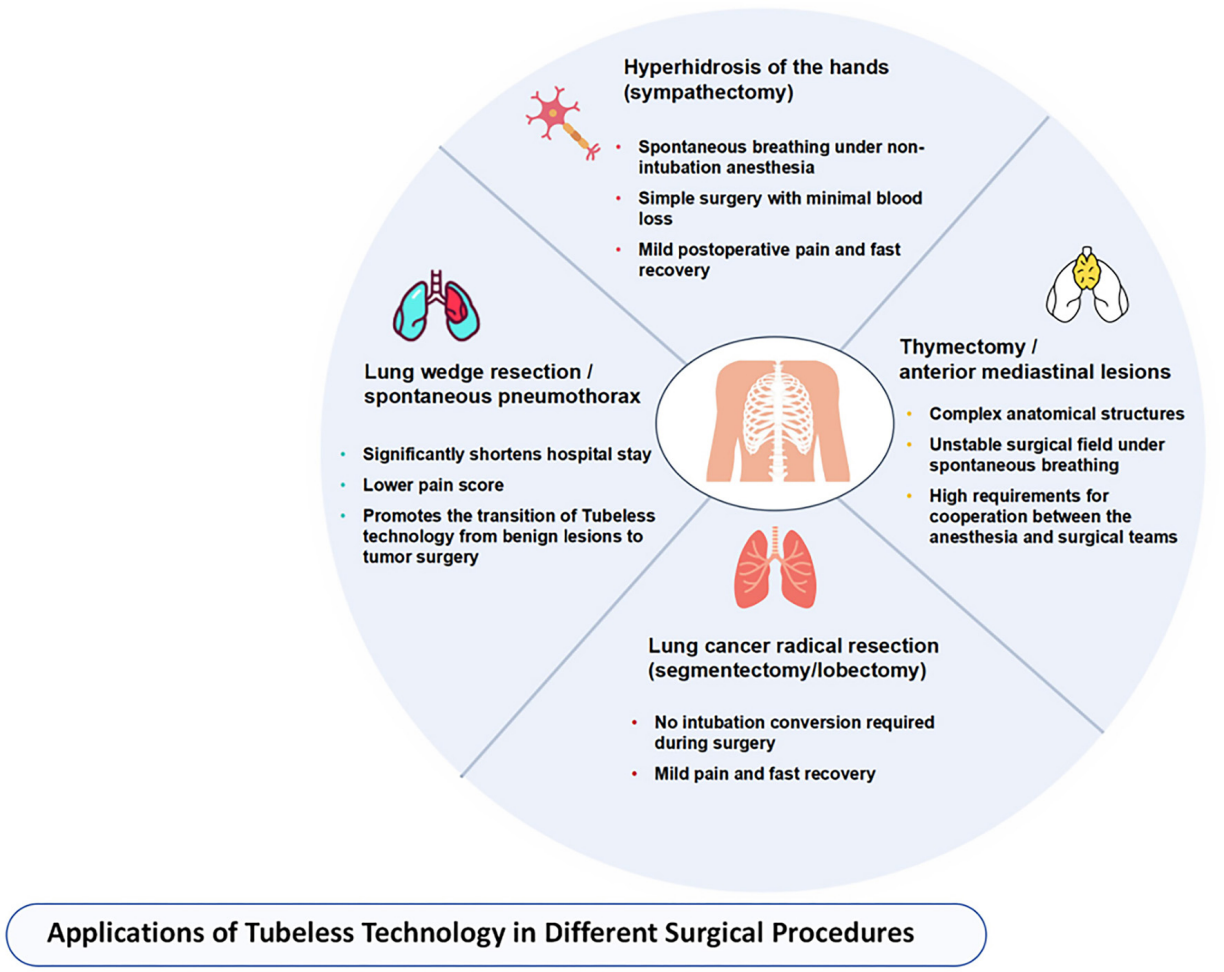
**Applications of tubeless uniportal VATS across thoracic procedures.** The diagram categorizes current clinical use into four representative settings—endoscopic thoracic sympathectomy for palmar hyperhidrosis, lung wedge resection for spontaneous pneumothorax, thymectomy/anterior mediastinal lesion resection, and anatomical lung cancer resection (segmentectomy/lobectomy)—reflecting the stepwise extension of tubeless practice from lower- to higher-complexity operations as perioperative protocols have matured. Abbreviation: VATS: Video-assisted thoracoscopic surgery.

With the refinement of non-intubated anesthesia and perioperative management protocols, tubeless uniportal thoracoscopic surgery has garnered increasing attention across various thoracic procedures. Its clinical application has progressively expanded from low-complexity surgeries, such as thoracic sympathectomy, to more technically demanding interventions, including pulmonary wedge resection, mediastinal tumor excision, and early-stage lung cancer [29, 30]. This section provides a categorized overview of its current clinical applications across diverse surgical scenarios. Applications of tubeless technology in various surgical procedures are illustrated in Figure 2.

### Primary palmar hyperhidrosis (thoracic sympathectomy)

Thoracic sympathectomy represents one of the earliest and most established applications of tubeless uniportal VATS [31]. It is frequently performed in younger patients with severe palmar sweating, which adversely affects their quality of life and social function [32]. Due to its superficial anatomy, limited dissection, and minimal bleeding risk, sympathectomy provides optimal conditions for the application of tubeless principles, characterized by non-intubated anesthesia, preserved spontaneous breathing, and drainage-free surgery [33]. From an anesthetic and perioperative management perspective, non-intubated TIVA combined with regional techniques, such as TPVB or ESPB, is commonly employed. Oxygenation is typically maintained using a laryngeal mask airway or high-flow nasal cannula, minimizing airway trauma while ensuring adequate ventilation and surgical exposure [34]. Additional intercostal nerve blocks or local infiltration with long-acting anesthetics may be utilized to further optimize postoperative analgesia. Clinical evidence consistently supports the feasibility and safety of tubeless endoscopic thoracic sympathectomy. In a large clinical series, Shao et al. [31] reported 172 patients undergoing tubeless sympathectomy with no intraoperative conversions, minimal postoperative pain (median visual analog scale score of 2 on postoperative day 0), and a median length of hospital stay of 1 day. Moreover, a recent meta-analysis comparing non-intubated and conventional intubated VATS demonstrated lower postoperative pain scores and improved early postoperative recovery in the non-intubated group [35]. Furthermore, Majeed et al. [36] reported sustained improvements in quality of life and high patient satisfaction during follow-up in a large cohort undergoing single-port endoscopic thoracic sympathectomy.

Overall, thoracic sympathectomy constitutes a highly standardized and low-risk setting for the application of tubeless uniportal VATS. The predictable anatomy, limited physiological disturbance, and favorable perioperative outcomes observed in this procedure make it a critical reference model for the gradual extension of tubeless techniques to more complex thoracic surgical interventions.

### Pulmonary wedge resection / spontaneous pneumothorax surgery

Spontaneous pneumothorax and bullous lung disease are among the most common benign indications for thoracic surgery, representing a logical next step in the application of tubeless uniportal VATS [37]. These conditions typically involve peripheral lesions and limited resection margins, providing favorable anatomical conditions for non-intubated and selectively drain-free thoracoscopic techniques [38]. Accordingly, tubeless pulmonary wedge resection is generally performed in young and otherwise healthy patients with preserved pulmonary function and without extensive pleural adhesions or emphysema.

From a technical standpoint, successful tubeless wedge resection relies on meticulous intraoperative hemostasis, effective prevention and management of air leaks, and confirmation of complete lung re-expansion without routine postoperative chest drainage [39]. Careful patient selection and intraoperative assessment are critical to ensuring procedural safety under spontaneous ventilation conditions. Clinical evidence supports the feasibility of this approach in selected patients. In a representative clinical series, Li et al. [38] reported that tubeless uniportal thoracoscopic surgery for pneumothorax and pulmonary bullae was feasible and safe, with a mean postoperative hospital stay of approximately 3.5 days and no major perioperative complications. These findings suggest that tubeless wedge resection can be reliably performed when strict selection criteria and standardized operative principles are applied.

Beyond its immediate clinical role, pulmonary wedge resection serves as a crucial transitional procedure in the gradual adoption of tubeless techniques. Its relatively standardized operative workflow, limited extent of resection, and manageable intraoperative variability make it an ideal platform for gaining experience in non-intubated anesthesia, air-leak control, and postoperative monitoring [40]. Continued refinement of perioperative management strategies may allow tubeless uniportal VATS in this context to facilitate broader acceptance of tubeless concepts and support their application in more complex thoracic procedures [17].

### Thymoma resection/anterior mediastinal lesions

While tubeless uniportal VATS has been well established for selected benign thoracic procedures, its use in anterior mediastinal tumors, such as thymoma, remains limited and primarily exploratory [41]. The anatomical proximity of the anterior mediastinum to vital structures, including the great vessels and pericardium, coupled with the confined operative space, presents distinct technical challenges for non-intubated uniportal thymectomy within a tubeless-oriented framework under spontaneous ventilation. Consequently, clinical experience has predominantly been confined to carefully selected patients with small, well-circumscribed early-stage thymomas (Masaoka stage I–II).

From a technical and anesthetic perspective, maintaining a stable surgical field during spontaneous breathing is a significant concern in non-intubated uniportal thymectomy. Diaphragmatic motion and mediastinal excursion can compromise visualization and hinder precise dissection, particularly near major vascular structures. To address these challenges, specialized centers have adopted strategies such as optimized patient positioning, customized curved instruments, and enhanced endoscopic visualization techniques. Comprehensive preoperative imaging evaluation and a detailed understanding of thymic and perithymic vascular anatomy are essential to minimize intraoperative risks [17].

Anesthetic management is critical for the safe execution of non-intubated uniportal thymectomy within a tubeless-oriented framework. Deep total intravenous anesthesia with propofol and remifentanil, combined with thoracic paravertebral or erector spinae plane blocks, is commonly utilized [42]. Controlled respiratory suppression, including brief apnea or assisted ventilation during critical procedural moments, is often employed to facilitate accurate dissection. Successful execution of these procedures necessitates close coordination between surgical and anesthesia teams to ensure airway security, hemodynamic stability, and optimal operative exposure [43].

Clinical evidence supporting tubeless or tubeless-related thymectomy remains limited but encouraging in carefully selected early-stage patient cohorts. In a representative case series, Liu et al. [44] reported on ten patients with early-stage thymoma associated with myasthenia gravis who underwent non-intubated uniportal subxiphoid thoracoscopic extended thymectomy. All procedures were completed successfully without conversion to intubated anesthesia or thoracotomy, and no major perioperative complications were observed. Although postoperative chest drainage was employed in this series, postoperative pain was generally mild, with visual analog scale (VAS) scores ranging from 1–3, indicating the technical feasibility of non-intubated uniportal thymectomy in specialized settings.

Overall, while the current evidence base is limited, available reports suggest that non-intubated uniportal thymectomy within a tubeless-oriented framework is both feasible and safe for carefully selected patients when performed in experienced centers. The technical complexity, restricted indications, and dependence on advanced anesthetic-surgical coordination highlight the necessity for cautious patient selection and the accumulation of further clinical experience before broader adoption is considered.

### Radical resection of lung cancer (lobectomy, segmentectomy)

The widespread implementation of low-dose computed tomography screening has led to increased detection of early-stage non-small cell lung cancer (NSCLC), fostering interest in extending tubeless uniportal VATS to anatomical lung resections [45]. Currently, its application is largely restricted to carefully selected patients with stage I disease, small peripheral tumors, and no radiological or intraoperative evidence of lymph node involvement, making rigorous patient selection essential for oncologic safety [46].

Compared to limited resections for benign disease or small pulmonary nodules, anatomical lung resection under tubeless conditions presents significantly greater technical and oncologic challenges. Although non-intubated and tubeless strategies have been increasingly explored in wedge resection and other sublobar procedures, dedicated clinical series focusing specifically on completely tubeless uniportal segmentectomy or lobectomy remain scarce, with most evidence stemming from small, single-center experiences or mixed procedural cohorts.

From a technical perspective, preventing and managing intraoperative air leakage is a critical challenge in tubeless anatomical lung resection. Strategies such as meticulous water seal testing, selective use of biological sealants, and confirmation of postoperative lung re-expansion through bedside ultrasound or chest radiography have been adopted. Some centers implement short-term “tubeless observation protocols” for early detection of complications [47, 48]. Concurrently, expert consensus statements and technical reviews underscore the importance of patient selection, air-leak control, and readiness for prompt conversion when necessary [49, 50], acknowledging that these sources provide conceptual guidance rather than primary patient-level outcome data.

From an oncologic perspective, the extension of tubeless uniportal VATS to anatomical lung resection necessitates cautious evaluation. Adequate lymph node dissection and sufficient resection margins are paramount for curative lung cancer surgery; however, both may be more technically demanding under spontaneous ventilation and limited operative exposure, particularly during complex hilar procedures or segmentectomy. Furthermore, the learning curve associated with tubeless uniportal anatomical resection should not be underestimated, as current experience primarily derives from high-volume centers, and early adoption may correlate with higher conversion rates or prolonged operative times [51].

In summary, while preliminary experience indicates that tubeless-oriented uniportal approaches may be technically feasible in carefully selected cases of early-stage lung cancer, the current evidence base remains limited. Although a randomized clinical trial has evaluated minimally invasive lung surgery using completely or partially tubeless protocols [14], robust large-scale multicenter randomized controlled trials specifically addressing completely tubeless uniportal anatomical resections—particularly lobectomy—are still lacking. Further well-designed prospective studies are required to validate oncologic safety, reproducibility, and broader clinical applicability. The currently available primary clinical evidence supporting tubeless uniportal VATS, predominantly in sympathectomy and sublobar procedures, is summarized in Table 1.

**Table 1:** Summary of representative clinical case series on tubeless uniportal VATS

**Procedure**	**Author (year)**	**Study design**	**Sample size**	**Anesthesia**	**Chest drain**	**Key outcomes**
ETS	Shao et al. (2022) [31]	Case series	172	SVA / non-intubated anesthesia	No	POD0 VAS (median) 2; LOS (median) 1 day; no conversion to intubated anesthesia
PSP / Wedge Resection	Li et al. (2019) [38]	Case series	18	Spontaneous breathing	No	Feasible and safe; mean LOS 3.5 days; no major complications
Thymoma	Liu et al. (2021) [44]	Case series	10	TIVA + LMA	Yes (bilateral small-bore catheter drainage, 3–5 days)	Feasible in selected patients; no conversion; low postoperative pain (VAS 1–3)
SPN / Wedge resection	Li et al. (2017) [61]	Case series	34	Spontaneous ventilation (non-intubated)	No	No conversion; VAS 2 ± 1; LOS 1 ± 1 day
Peripheral lung nodules / Wedge resection	Yang et al. (2017) [62]	Case series	30	Non-intubated spontaneous ventilation	No	No conversion; POD1 VAS 1.0 ± 0.8; LOS 3.1 ± 0.7 days

Note: “Tubeless” refers to the intentional exclusion of routine postoperative chest drainage; selective drainage, when clinically warranted, is reported accordingly. Abbreviations: SVA: Spontaneous ventilation anesthesia; TIVA: Total intravenous anesthesia; LMA: Laryngeal mask airway; ETS: Endoscopic thoracic sympathectomy; PSP: Primary spontaneous pneumothorax; SPN: Solitary pulmonary nodule; VATS: Video-assisted thoracoscopic surgery.

## Advantages and challenges of tubeless uniportal technology

With the growing clinical adoption of tubeless uniportal thoracoscopic surgery across various thoracic procedures, there is an increasing focus on its therapeutic value and translational potential. Drawing from clinical experiences in sympathectomy, pulmonary wedge resection, thymectomy, and early-stage lung cancer resection, this section summarizes the key benefits and implementation challenges associated with this technique.

### Clinical advantages: Promoting postoperative rehabilitation and optimizing patient experience

The primary clinical advantage of tubeless uniportal VATS lies in its capacity to minimize perioperative invasiveness, thereby enhancing postoperative recovery and patient comfort [52]. Non-intubated anesthesia circumvents complications associated with tracheal intubation, such as sore throat, bronchospasm, and atelectasis. The elimination of chest drains significantly reduces pain related to intercostal irritation and facilitates earlier mobilization, which in turn promotes the recovery of pulmonary function [53]. Moreover, ERAS-oriented, opioid-sparing analgesic strategies—often implemented alongside tubeless protocols—may mitigate opioid-related side effects and streamline nursing care.

These advantages are substantiated by various clinical studies. For instance, Wang et al. [54] reported lower postoperative pain scores during the early recovery phase and a trend toward shorter hospital stays in patients undergoing non-intubated or tubeless segmentectomy. Similarly, Pompeo [17] noted early postoperative ambulation and reduced analgesic requirements following tubeless wedge resection. These findings align with the principles of ERAS and underscore the potential role of tubeless VATS in enhancing postoperative rehabilitation.

### Practical limitations of technology and promotion

Despite its clinical promise, the broader implementation of tubeless uniportal VATS is constrained by several technical and systemic challenges. First, the technique is inherently complex. Non-intubated anesthesia necessitates the maintenance of stable spontaneous ventilation, while thoracoscopic manipulation can be affected by diaphragmatic movement and fluctuating visual fields. These dynamics heighten the demand for precise surgical coordination, particularly during procedures involving hilar dissection or bronchial division [5].

Second, successful execution relies heavily on the experience and teamwork of both the surgical and anesthesia teams. Ensuring adequate analgesia, oxygenation, and field exposure under spontaneous breathing conditions requires a high level of intraoperative collaboration. Furthermore, the absence of standardized clinical pathways—including protocols for patient selection, air leak management, and postoperative drainage strategies—continues to hinder widespread adoption. The current evidence base is also limited; most published studies are retrospective, single-center analyses with small sample sizes. The lack of multicenter prospective randomized controlled trials (RCTs) has restricted the formulation of consensus guidelines and evidence-based recommendations. To facilitate broader adoption, future efforts should concentrate on establishing unified technical standards, multidisciplinary training programs, and high-quality clinical trials. A comparison of tubeless and conventional VATS techniques is presented in Table 2, highlighting their respective clinical features and limitations.

**Table 2:** Comparative analysis of tubeless uniportal VATS versus conventional VATS

**Parameter**	**Tubeless uniportal VATS**	**Conventional VATS**
Anesthesia	Non-intubated, spontaneous breathing	Intubated general anesthesia
Chest drain	Often omitted (reported in selected series)	Routinely placed
Postoperative pain	Generally lower postoperative pain reported (reported in selected series/centers)	Generally higher postoperative pain reported
Recovery time	Shorter hospital stay; early ambulation (in carefully selected patients)	Longer hospitalization
Patient selection	Strict; low-risk cases	Broader inclusion criteria
Technical complexity	High; requires experienced team	Moderate; more standardized
Evidence level	Mainly derived from case series and limited randomized evidence	Supported by multiple randomized trials and meta-analyses

Abbreviation: VATS: Video-assisted thoracoscopic surgery.

It is important to note that the existing evidence supporting tubeless uniportal VATS is heterogeneous in study design and clinical context. Most available studies are retrospective or observational series with small sample sizes, and only a limited number of randomized or comparative trials have been reported. Moreover, favorable outcomes are primarily derived from high-volume centers with substantial expertise in non-intubated thoracic anesthesia and advanced uniportal techniques, introducing potential selection and center-experience bias. Thus, existing data should be interpreted with caution when extrapolating to broader clinical settings.

In addition to evidence-related limitations, tubeless uniportal VATS is associated with specific intraoperative risks and failure-to-proceed scenarios. Hypoxemia due to hypoventilation or prolonged lung collapse, inadequate suppression of the cough reflex, hemodynamic instability, and unexpected bleeding may necessitate conversion [55]. Both anesthesiologic and surgical learning curves play a critical role in mitigating these risks, as early adoption is often linked to higher conversion rates and longer operative times. These considerations emphasize the necessity of structured training, stepwise implementation, and clearly defined conversion criteria.

### Patient selection: inclusion criteria and contraindications

Given the critical role of patient selection in the safety and feasibility of tubeless uniportal VATS, commonly reported inclusion criteria and contraindications merit summarization. Suitable candidates typically exhibit adequate cardiopulmonary reserve, stable respiratory function, and a low risk of airway compromise, and they undergo anatomically straightforward procedures with limited bleeding risk under spontaneous ventilation. Relative contraindications include obesity, moderate chronic obstructive pulmonary disease, anticipated difficult airway management, extensive pleural adhesions, or complex hilar dissection [56]. Absolute contraindications commonly cited include severe hypoxemia, unstable cardiopulmonary disease, high aspiration risk, anticipated massive bleeding, or inability to ensure timely conversion to intubated anesthesia. These considerations underscore the importance of meticulous patient selection and appropriate institutional experience [57].

In clinical practice, clear conversion criteria are essential to ensure patient safety during tubeless uniportal VATS. Commonly reported triggers for conversion to endotracheal intubation include persistent hypoxemia despite optimization of spontaneous ventilation, uncontrolled hypercapnia, excessive patient movement or cough compromising surgical safety, hemodynamic instability, and unexpected major bleeding [58]. The inability to maintain a stable operative field under spontaneous breathing is also frequently cited. Indications for chest drain placement during or after tubeless procedures commonly include significant or persistent air leakage, incomplete lung re-expansion, or intraoperative bleeding requiring postoperative monitoring. Importantly, timely conversion or drain placement should be regarded as a safety measure rather than a procedural failure, with predefined conversion thresholds widely emphasized in expert reports [55].

## Outlook and future development direction

Tubeless uniportal thoracoscopic surgery represents a significant advancement in minimally invasive thoracic procedures, demonstrating clinical feasibility and early success across multiple surgical indications. However, transitioning from experimental application to widespread adoption necessitates further development in several areas, including technical standardization, intelligent assistance, evidence accumulation, and integration with perioperative care frameworks. This section delineates four key directions for future advancement.

### Technical standardization and clinical guideline construction

Currently, no unified protocols exist for patient selection, anesthesia strategies, intraoperative management, or postoperative care in tubeless uniportal VATS. Variability across centers in drainage management, ERAS-oriented pain control methods, and contingency plans significantly affects reproducibility and generalizability. Establishing standardized procedural pathways—stratified by surgical complexity—would facilitate broader implementation. It is recommended that academic organizations collaborate to publish consensus-based clinical guidelines defining procedural classifications, intraoperative milestones, and conversion thresholds, supported by training platforms and simulation-based education [10].

### Integration of artificial intelligence and surgical navigation

Intraoperative control remains one of the most technically demanding aspects of tubeless thoracic surgery, particularly under spontaneous ventilation. Emerging technologies such as artificial intelligence (AI), augmented reality (AR), and intraoperative imaging offer potential solutions. For instance, three-dimensional segmentation algorithms and vascular mapping derived from preoperative CT imaging can enhance surgical planning. AI-guided risk prediction models may eventually aid real-time decision-making based on intraoperative respiratory fluctuations or bleeding risk [59, 60]. These tools are still under development, necessitating future studies to validate their utility in tubeless contexts.

### The need for multicenter prospective studies and randomized controlled trials

Most existing data on tubeless VATS originate from single-center, retrospective case series with limited sample sizes. Although randomized evidence is beginning to emerge for minimally invasive lung surgery utilizing tubeless protocols, robust multicenter RCTs specifically focused on completely tubeless uniportal procedures are scarce, limiting the generalizability of current findings. To strengthen the evidence base, prospective multicenter RCTs should systematically assess efficacy, safety, and long-term outcomes across specific procedures, including segmentectomy, thymectomy, and lobectomy. Relevant endpoints should encompass postoperative complication rates, pulmonary function recovery, immune modulation, and quality-of-life measures. Additionally, registry-based real-world studies may complement RCT data by providing insights into economic outcomes and scalability in routine clinical practice.

### Integration with the ERAS concept: Creating a comprehensive minimally invasive chain from preoperative to intraoperative to postoperative

The fundamental objectives of tubeless technology align closely with the ERAS concept. In the future, tubeless uniportal VATS may be integrated into ERAS-aligned thoracic surgical pathways as a key intraoperative strategy rather than a standalone perioperative framework. ERAS-oriented care encompasses preoperative optimization (e.g., nutrition, psychological preparation, pulmonary training), intraoperative management (e.g., non-intubated anesthesia, spontaneous ventilation, selective omission of drainage), and postoperative rehabilitation (e.g., analgesia optimization, early mobilization, individualized discharge criteria). By incorporating tubeless principles into ERAS-based pathways, it may be possible to further shorten hospital stays, reduce perioperative risks, and enhance patient-centered outcomes.

In summary, the continued evolution of tubeless uniportal thoracoscopic surgery will depend on interdisciplinary collaboration, technological innovation, and rigorous clinical validation. These efforts are essential for transitioning the technique from a niche approach to a standardized component of modern thoracic surgery.

### Stepwise implementation and training pathway

Given the reliance of tubeless uniportal VATS on multidisciplinary experience, a stepwise implementation strategy is advisable. Initial adoption is generally recommended for low-risk, standardized procedures such as thoracic sympathectomy, allowing teams to become acclimated to non-intubated anesthesia, spontaneous ventilation, and conversion protocols. As experience increases, progression to pulmonary wedge resection or spontaneous pneumothorax surgery may be considered, introducing air-leak management and lung re-expansion assessment while maintaining limited procedural complexity. Mediastinal procedures, such as thymectomy, require further refinement of anesthetic-surgical coordination to ensure field stability under spontaneous breathing. Anatomical lung resections, including segmentectomy and select lobectomy, should be reserved for centers with substantial expertise, where strict patient selection, predefined conversion criteria, and close intraoperative collaboration are established. Such a staged pathway may facilitate the safe adoption and gradual expansion of tubeless uniportal VATS.

### Limitations

Several limitations of this review should be acknowledged. As a narrative review, the synthesis of literature was qualitative rather than systematic, and no formal study-level quality appraisal or quantitative comparison was performed. The review was confined to English-language publications, potentially leading to the omission of relevant studies. Furthermore, significant heterogeneity exists in procedural definitions, patient selection criteria, and interpretations of “tubeless” protocols across studies, which limits direct comparability. Lastly, the available evidence predominantly stems from small, single-center observational studies, and potential publication bias toward favorable outcomes cannot be discounted. These limitations should be considered when interpreting the findings and underscore the need for standardized reporting and higher-quality prospective studies.

## Conclusion

Tubeless uniportal thoracoscopic surgery has demonstrated favorable feasibility, safety, and early recovery benefits in select thoracic procedures, particularly sympathectomy, wedge resection, and early-stage lung cancer. As an advanced minimally invasive technique, it offers a patient-centered option that minimizes perioperative trauma. Broader adoption will require standardized clinical pathways, intelligent assistance, and multicenter validation to ensure safe and evidence-based application in more complex operations.
